# Early Identification of High-Risk TIA or Minor Stroke Using Artificial Neural Network

**DOI:** 10.3389/fneur.2019.00171

**Published:** 2019-03-01

**Authors:** Ka Lung Chan, Xinyi Leng, Wei Zhang, Weinan Dong, Quanli Qiu, Jie Yang, Yannie Soo, Ka Sing Wong, Thomas W. Leung, Jia Liu

**Affiliations:** ^1^Department of Medicine and Therapeutics, Prince of Wales Hospital, The Chinese University of Hong Kong, Hong Kong, China; ^2^Shenzhen Research Institute, The Chinese University of Hong Kong, Shenzhen, China; ^3^Shenzhen Institutes of Advanced Technology, University of Chinese Academy of Sciences, Shenzhen, China; ^4^Department of Neurology, The Second Affiliated Hospital of Guangzhou Medical University, Guangzhou, China

**Keywords:** transient ischemic attack, minor stroke, artificial neural network, risk stratification, prognosis

## Abstract

**Background and Purpose:** The risk of recurrent stroke following a transient ischemic attack (TIA) or minor stroke is high, despite of a significant reduction in the past decade. In this study, we investigated the feasibility of using artificial neural network (ANN) for risk stratification of TIA or minor stroke patients.

**Methods:** Consecutive patients with acute TIA or minor ischemic stroke presenting at a tertiary hospital during a 2-year period were recruited. We collected demographics, clinical and imaging data at baseline. The primary outcome was recurrent ischemic stroke within 1 year. We developed ANN models to predict the primary outcome. We randomly down-sampled patients without a primary outcome to 1:1 match with those with a primary outcome to mitigate data imbalance. We used a 5-fold cross-validation approach to train and test the ANN models to avoid overfitting. We employed 19 independent variables at baseline as the input neurons in the ANN models, using a learning algorithm based on backpropagation to minimize the loss function. We obtained the sensitivity, specificity, accuracy and the c statistic of each ANN model from the 5 rounds of cross-validation and compared that of support vector machine (SVM) and Naïve Bayes classifier in risk stratification of the patients.

**Results:** A total of 451 acute TIA or minor stroke patients were enrolled. Forty (8.9%) patients had a recurrent ischemic stroke within 1 year. Another 40 patients were randomly selected from those with no recurrent stroke, so that data from 80 patients in total were used for 5 rounds of training and testing of ANN models. The median sensitivity, specificity, accuracy and c statistic of the ANN models to predict recurrent stroke at 1 year was 75%, 75%, 75%, and 0.77, respectively. ANN model outperformed SVM and Naïve Bayes classifier in our dataset for predicting relapse after TIA or minor stroke.

**Conclusion:** This pilot study indicated that ANN may yield a novel and effective method in risk stratification of TIA and minor stroke. Further studies are warranted for verification and improvement of the current ANN model.

## Introduction

The prevalence of transienti ischemic attack (TIA) is estimated to be 103.3 per 100,000 in the Chinese population (1). Although TIA may be regarded as a “benign” cerebrovascular event, subsequent stroke could be disabling. Studies conducted over 15 years ago reported that 12–20% of TIA or minor stroke patients would have a recurrent stroke within 3 months (2). The risk of stroke recurrence in such patients has been declining over the past decade partly due to advances in the stroke service system −5.1% of TIA or minor stroke patients in hospitals with dedicated TIA/minor stroke service systems had a recurrent stroke within 1 year in the recently published TIAregistry.org Project, but there have been concerns that the recurrent rate is probably higher in routine clinical practice (3). In the Clopidogrel in High-Risk Patients with Acute Nondisabling Cerebrovascular Events (CHANCE) trial, 10.0% of 5,170 minor stroke patients or TIA patients with an ABCD2 score≥ 4 recruited from 114 hospitals of different levels in China had a recurrent stroke at 3 months, despite of early-initiated dual or mono antiplatelet treatment (4).

A few risk scores have been developed to identify high-risk TIA or minor stroke patients, for instance, the ABCD^2^ score, which has been commonly used in research and in clinical practice. Patients with an ABCD^2^ score≥4 are generally considered as high-risk patients (5, 6). However, recent studies indicated that ABCD^2^ score may not reliably differentiate TIA or minor stroke with mimics, or those at high or low risk of recurrent stroke (7). Moreover, those with a ABCD^2^ score <4 and ≥4 could have similar risks of recurrent stroke at 3 months (8). Other factors have been considered to supplement the ABCD^2^ score, for instance, presence of new infarct(s) and carotid arterial stenosis and dual TIA, to form the ABCD^3^-I score (9). These new scoring systems have been well validated in other populations, reporting the c-statistics of 0.60–0.64 in predicting the recurrent stroke within 3 month following TIA. However, new scores such as ABCD^3^-I score have not been recommended for risk stratification in such patients by the guidelines by far (10, 11).

**Figure 1:**
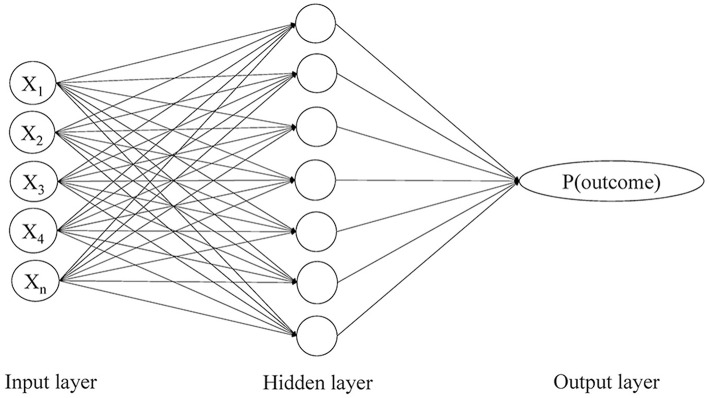
The three-layer perceptron artificial neural network model showing input, hidden and output layers and nodes with feed forward links. P(outcome) refers to the probability of an outcome.

In the current study, we aimed to use a novel method to predict the risk of stroke recurrence in TIA or minor stroke patients, which was the artificial neural network (ANN) technique. It is a commonly used machine learning algorithm to form a diagnostic or risk prediction model, which typically consists of three layers of neurons, an input layer of independent variables, a hidden layer with no real-world meaning but allowing nonlinear interactions among the input variables, and an output layer for the probability of an outcome (Figure 1). Advantages of ANN over conventional statistical methods in forming a risk prediction model lie in that it detects interactions between the input variables that commonly exist in clinical studies, and that it takes into account the weights of input variables in their correlations with the outcome (12, 13).

Previous studies have used ANN to diagnose acute myocardial infarction and stroke, and to predict mortality in ischemic stroke, intracerebral hemorrhage, traumatic brain injury, etc., which demonstrates improved accuracy against conventional methods in most circumstances (14–18). However, to our knowledge, ANN had not been applied in predicting the risk of recurrence following a TIA or minor stroke. Therefore, in this pilot study, we developed and tested ANN models for risk stratification of TIA or minor stroke. In addition, other algorithms for machine learning such as Support Vector Machine (SVM) and Naïve Bayes classifiers (NBC) have also been utilized in medical research (19, 20). For instance, SVM has been extensively applied in diagnosis of a disease or classification of groups with certain features based on imaging data (20). The NBC algorithm was able to diagnose carpel tunnel syndrome with the highest detection rate among four machine learning methods (21). Thus, in the current cohort, we also compared the performance of ANN with SVM and NBC in risk stratification of TIA and minor stroke patients.

## Materials and Methods

### Study Design and Subjects

Consecutive patients with acute TIA or minor ischemic stroke presenting at a tertiary hospital between January 2004 and December 2005 were recruited. TIA was defined as a transient episode of neurological dysfunction caused by focal brain ischemia, which completely resolved within 24 h. A minor ischemic stroke was diagnosed as sudden onset of neurological deficits caused by brain ischemia lasting longer than 24 h, with admission NIHSS score of 0–3. Diagnosis of TIA and minor ischemic stroke was made by the neurologists in charge. Stroke or TIA mimics such as toxic metabolic syndrome, seizure, migraine, demyelinating disorders, drug ingestion were excluded (22). We collected patients' characteristics at baseline as detailed below, including well-established factors readily available in clinical practice that might be associated with stroke recurrence. TIA and minor stroke patients were regularly followed up at the outpatient clinic, when recurrent cerebral ischemic events and other events were recorded. The primary outcome was defined as recurrent ischemic stroke within 1 year, as confirmed with CT or MR imaging, or diagnosed by the neurologist in charge. We developed and tested ANN models based on patients' characteristics to predict the primary outcome. We also conducted conventional statistical analyses for independent predictors for the primary outcome. The study was approved by the Joint Chinese University of Hong Kong–New Territories East Cluster Clinical Research Ethics Committee (The Joint CUHK-NTEC CREC).

### Data Collection

We collected demographic characteristics (sex, age) and vascular risk factors (smoking, hypertension, diabetes mellitus, dyslipidemia, prior TIA or ischemic stroke, atrial fibrillation, ischemic heart disease) at baseline. We also collected certain clinical characteristics including systolic and diastolic blood pressure at admission, National Institutes of Health Stroke Scale (NIHSS) score at admission, premorbid modified Rankin Scale (mRS) score, symptom duration (for transient deficits) and symptom type (unilateral weakness and slurring speech). We also gathered medications prescribed at discharge, such as antiplatelets, antihypertensive, anticoagulants, antidiabetics and statins.

We also collected neuroimaging features including new infarct(s) on brain CT or magnetic resonance imaging (MRI), and findings by cerebrovascular workup including presence of extra-/intra-cranial arterial stenosis. Extracranial arterial stenosis was defined as at least 50% narrowing of the internal carotid artery or vertebral artery lumen on carotid duplex ultrasound, CT or MR angiography, or digital subtraction angiography, by the NASCET method (23). Intracranial artery stenosis was defined as at least 50% narrowing of middle cerebral artery, anterior cerebral artery, posterior cerebral artery, intracranial segment of internal carotid artery and vertebrobasilar arteries in transcranial Doppler, cerebral CT or MR angiography, or digital subtraction angiography, using WASID method (24). We defined large artery stenosis as either extracranial arterial stenosis or intracranial arterial stenosis.

### Training and Testing in ANN

We employed a three-layer multilayer perceptron (MLP) model in this study, a most common type of ANN. It was composed of an input layer of 19 independent variables (Supplemental Table S1), a hidden layer with a certain number of neurons that were adjusted through training, and an output layer representing the probability of the primary outcome (Figure 1). A backpropagation algorithm was used to minimize the loss function by iteratively updating the weights between the neurons and thus maximize the predictive power of the ANN model for the primary outcome. Loss function represented the inconsistency between the predictive and actual values. Within each iteration of the backpropagation algorithm, the partial derivatives of the loss function with respect to each weight were propagated backward from the output layer and passed through the hidden layer, which eventually adjusted all the weights back to the input neurons.

**Figure 2:**
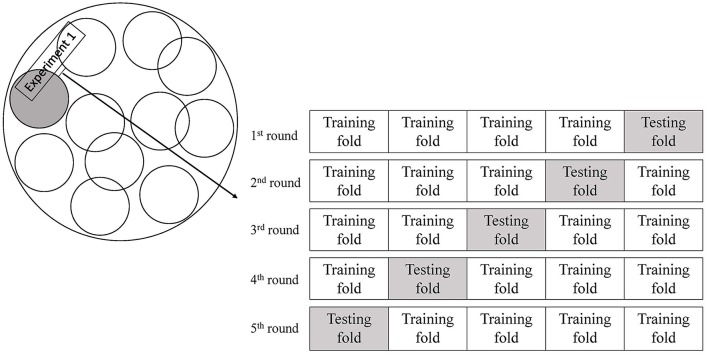
Schematic representation of 10 experiments with 5-fold cross-validation.

The study cohort was imbalanced in the numbers of patients with or without a primary outcome. This may cause biased prediction toward the no-recurrence group. Therefore, we randomly selected the same number of patients without a primary outcome as patients with a primary outcome for the training and testing (1:1 matched). In view of the relatively small sample size, we used a 5-fold cross-validation approach to train and test the ANN models (Figure 2), to avoid overfitting of the models (25, 26). The dataset was randomly partitioned into 5 folds, and we performed 5 rounds of training and testing of the ANN models. In each round of the experiments, 4 folds were the training subsets and the remaining subset was retained to test the ANN model. Each of the 5 subsets was only used once as the testing set in the 5-fold cross-validation process. We implemented the training and testing procedures for the ANN models in Matlab. We trained and tested ANN models with 4, 6, 8, 10, and 12 hidden neurons, respectively, and finalized the number of hidden neurons when the ANN model reached a minimal loss function in each round of the cross-validation experiments. We repeated such cross-validation procedures for 10 times (10 Experiments, Figure 2), each time with a new group of randomly selected patients without a primary outcome 1:1 matched with patients with a primary outcome.

For continuous independent variables, data normalization was required to speed up the gradient, thus to find the optimal solution. Shapiro-Wilk test was performed to determine whether a continuous independent variable was normally distributed. If not normally distributed, we scaled the data to a range of 0–1 before entering an ANN model. To be efficient in achieving the minimization of the loss function, we employed an adaptive moment estimation (Adam) rate rather than the constant learning rate. In addition, we assigned a weight between −1 and +1 for the independent variables to speed up the learning process and escape local minima. We obtained the sensitivity, specificity, accuracy, and the c statistic of the ANN models developed in each of the 5 rounds of training and testing, in each of the 10 cross-validation experiments; we then computed the overall medians (interquartile range, IQR) of sensitivity, specificity, accuracy and c statistic of the models in all of the 50 rounds of training and testing.

### Training and Testing in SVM and NBC

For training the SVM models, we tried a few standard kernel functions, such as sigmoid, Gaussian, polynomial and linear kernel, and adjusted different values of the parameter C in the range of 0.1–10. We finally determined the kernel function and the value of the parameter C by the highest accuracy in predicting the test set.

Additionally, using the Naïve Bayesian equation to calculate the posterior probability for each class, we first computed the prior probability of each class P(c), of each predictor P(x), and the likelihood which is the probability of predictor given class P(x|c). The outcome of prediction was the class with the highest posterior probability, using the Maximum A Posteriori (MAP) estimation.

In accordance with the ANN, we also applied the 5-fold cross-validation approach for both SVM and NBC and repeated it for 10 times with randomly selected patients without a primary outcome to 1:1 match the patients with a primary outcome. The overall medians (IQR) of sensitivity, specificity, accuracy and c statistic were also calculated for SVM models and NBC. We used Kruskal–Wallis test to compare the overall medians of sensitivity, specificity, accuracy and c statistic among the three machine learning algorithms and *post-hoc* comparisons between any two of these algorithms.

### Other Statistical Analyses

In addition, we also conducted conventional statistical analyses for predictors for the primary outcome in the study cohort. Continuous variables were presented as medians (interquartile range [IQR]), whilst categorical variables were presented as numbers (percentage). For univariate comparisons between patients with and without the primary outcome, continuous variables were analyzed with independent *t*-tests or Mann–Whitney *U*-test, whilst categorical variables were analyzed with χ^2^-test or Fisher's exact test. To identify factors independently associated with the primary outcome, variables with *p* < 0.1 in univariate analyses were entered in a multivariate logistic regression model for further analysis. Odds ratio (OR) and 95% confidence interval (CI) were calculated. *P* < 0.05 were considered statistically significant. All the conventional statistical analyses were conducted in IBM SPSS Statistics version 22.0 (SPSS Inc., Chicago, IL, United States).

## Results

In total, 451 patients were recruited; 201 (44.6%) patients had a TIA and the remaining had a minor stroke as an index ischemic event. The median NIHSS was 1 (IQR 0–2). Forty (8.9%) patients had the primary outcome of recurrent ischemic stroke within 1 year. Twelve patients died within 1 year, three of which developed recurrent ischemic stroke before death; and the remaining nine patients died from other reasons. More of the patients with a primary outcome had a history of TIA (15 vs. 5.4%, *p* = 0.038), and extra- and/or intra-cranial large artery stenosis (62.5 vs. 35.8%, *p* = 0.001), compared with patients without a primary outcome event (Table 1). Other baseline characteristics or medications prescribed at discharge were not significantly different between those with and without a primary outcome in the study cohort (Table 1). History of TIA, large artery stenosis, atrial fibrillation and smoking were further analyzed in multivariate logistic regression to predict the primary outcome. Only presence of large artery stenosis (OR: 2.87; 95% CI: 1.45–5.67; *p* = 0.002) was significantly associated with recurrent ischemic stroke within 1 year following a TIA or minor stroke in multivariate analysis.

**Table 1:** Characteristics of patients with or without recurrent ischemic stroke within 1 year.

**Variables**	**Recurrence (*n* = 40)**	**No recurrence (*n* = 411)**	***P*-value**
Age, years	71 (60–74)	65 (55–73)	0.116
Male	20 (50.0)	231 (56.2)	0.451
History of hypertension	29 (72.5)	256 (62.3)	0.201
History of diabetes mellitus	13 (32.5)	123 (29.9)	0.735
History of dyslipidemia	21 (52.5)	252 (61.3)	0.276
History of AF	7 (17.5)	37 (8.9)	0.084
History of ischemic stroke	10 (25.0)	68 (16.5)	0.177
History of TIA	6 (15.0)	22 (5.4)	0.038
History of ischemic heart disease	4 (10.0)	39 (9.5)	0.916
Smoker	8 (20.0)	142 (34.5)	0.062
Unilateral weakness	25 (62.5)	226 (55.0)	0.213
Slurring speech	15 (37.5)	107 (26.0)	0.361
Symptom duration			0.355
≤10 min	3 (7.5)	63 (15.3)	
11–60 min	4 (10)	50 (12.2)	
>60 min	33 (82.5)	298 (72.5)	
Systolic blood pressure, mmHg	166 (146–182)	160 (143–180)	0.453
Diastolic blood pressure, mmHg	80 (71–97)	83 (74–94)	0.949
NIHSS at admission	1 (0.25–2)	1 (0–2)	0.893
Premorbid mRS	0 (0–0)	0 (0–0)	0.125
Large artery stenosis	25 (62.5)	147 (35.8)	0.001
New infarct	11 (27.5)	102 (24.8)	0.709
Antiplatelet(s)	29 (72.5%)	320 (77.9%)	0.916
Anticoagulant	0 (0.0%)	9 (2.2%)	1.000
Antihypertensives	19 (47.5%)	209 (50.9%)	0.851
Antidiabetics	6 (15.0%)	92 (22.4%)	0.409
Statins	16 (40.0%)	182 (44.3%)	0.984

*AF indicates atrial fibrillation; TIA indicates transient ischemic attack; NIHSS indicates National Institute of Health Stroke Scale; mRS indicates modified Rankin Scale*.

In each of the 10 experiments of developing and testing the ANN models with 5-fold cross-validation, 40 patients were randomly selected from those without a primary outcome to 1:1 match with the 40 patients with a primary outcome (Figure 2). The number of neurons in the hidden layer was finalized as 10, after testing 4, 6, 8, 10, and 12 hidden neurons in the models. The median sensitivity, specificity and accuracy of the ANN models was 75% (63.3–83.3%), 75% (62.5–83.3%), and 75% (68.8–76.6%), respectively. The median c statistic was 0.77 (0.68–0.84) (Table 2).

**Table 2:** Predictive perfrmance of ANN, SVM and NBC models.

Statistics^*^	**ANN**	**SVM**	***P*-value (ANN vs. SVM)**	**Naïve Bayes**	***P*-value (ANN vs. NBC)**	***P*-value (overall comparison)**
Sensitivity	75% (63.3–83.3%)	62.5% (50–62.5%)	<0.001	62.5% (50–75%)	0.001	<0.001
Specificity	75% (62.5–83.3%)	75% (50–87.5%)	0.081	75% (62.5–75%)	0.121	0.151
Accuracy	75% (68.8–76.6%)	62.5% (56.3–68.8%)	<0.001	62.5% (56.3–68.8%)	<0.001	<0.001
C statistic	0.77 (0.68–0.84)	0.63 (0.56–0.69)	<0.001	0.63 (0.56–0.69)	<0.001	<0.001

* *presented with medians (IQR)*.

*ANN indicates artificial neural network; SVM indicates support vector machine; NBC indaictaes Naïve Bayes classifier*.

After testing several kernel functions and values of parameter C, we found that the SVM model with the linear kernel and parameter C equaling to 1 was optimal among all the others. The median sensitivity, specificity and accuracy of the SVM models was 62.5% (50–62.5%), 75% (50–87.5%), and 62.5% (56.3–68.8%).

Moreover, we also calculated the posterior probability for each class based on the Naïve Bayesian equation, and selected the outcome with highest probability. The median sensitivity, specificity and accuracy of the NBC was 62.5% (50–75%), 75% (62.5–75%), and 62.5% (56.3–68.8%). The performance of ANN models in identifying patients with recurrent ischemic stroke was better than that of SVM and NBC algorithm (Table 2).

## Discussion

This pilot study demonstrated the feasibility of using ANN to predict the risk of recurrent stroke within 1 year after a TIA or minor stroke, based on parameters that are readily available in clinical practice. With a relatively small sample size and a smaller number of the primary outcome event, conventional univariate and multivariate analyses only identified the presence of cervico-cerebral large artery stenosis as an independent predictor for stroke recurrence within 1 year. However, the ANN models developed based on this study cohort showed moderate-to-good accuracy in predicting the primary outcome in comparison with SVM model and Naïve Bayes classifier, which suggested ANN as an alternative or even more effective approach for risk stratification of TIA or minor stroke.

Despite of the small sample size, presence of extra- and/or intra-cranial large artery stenosis was identified as an independent risk factor of recurrent stroke in the current study. This was consistent with relevant findings in the TIAregistry.org project and other previous studies. For instance, in the TIAregistry.org project, major brain imaging findings, including ≥1 acute ischemic lesion and ≥1 intra- and/or extra-cranial stenosis >50%, were associated with increased risk of stroke recurrence at 3 months or 1 year after a TIA or minor stroke (3). Particularly in subjects recruited from Asia in the TIAregistry.org project, presence of intracranial stenosis tended to increase the 1-year stroke risk, independent of other confounding factors (*p* = 0.09) (27). Therefore, the current study reinforced intracranial stenosis as a strong risk factor for stroke recurrence in Asians.

ANN models developed in the current study showed moderate-to-good accuracy in predicting the primary outcome, while we used a 5-fold cross-validation approach to avoid overfitting of the models. Previous studies also found ANN models accurate and effective in differentiating cerebral ischemia from stroke mimics, and in predicting mortality in patients with intracerebral hemorrhage, etc. (14, 17). As mentioned above, ANN possessed advantages over conventional statistical methods in forming a risk prediction or diagnostic model. ANN could detect complex nonlinear relationships between independent and dependent variables and assign weights to the independent variables in their associations with the outcome, thus enhancing the model fit as compared with logistic regression methods (13). ANN could also take into account possibly complex interactions between the independent variables, (28) which commonly exist in clinical scenarios, e.g., interactions between age and presence of the vascular risk factors. Moreover, in the era of precision medicine, simple dichotomization of a factor as a continuous variable in nature (e.g., age and blood pressure) in conventional scoring systems may not accurately reflect the effects of these variables in determining the risk of stroke recurrence, while the ANN approach could accommodate variables as they are in the risk prediction models.

Our results showed that the ANN outperformed the SVM and NBC. For the SVM, we tested the standard kernel functions and the best accuracy was achieved with merely 62.5% by a linear kernel. This suggests that the standard nonlinear kernel functions we tested might not be appropriate for projecting the data into a space where they can be classified by an SVM. Finding a better kernel function for this particular problem is however not intuitive and is not in the scope of this study. In contrast, the major advantage of the proposed ANN is that projecting the data into a space for classification is driven by the data. Thus, there is no need to pre-define a kernel function. For the NBC, the assumption of independence between the input variables might not be well satisfied for this study. This can negatively affect the accuracy of the NBC. Though this can possibly be improved by carefully selecting the input variables, we however did not try this procedure in order to show the ANN is an end-to-end approach that does not require data pre-selection.

The present study had several limitations. It was a retrospective single-center study with data collected years ago, but this pilot study indicated potential application of ANN in risk stratification of TIA and minor stroke patients. In addition, the study cohort was inevitably imbalanced in view of the numbers of patients with and without the primary outcome. We mitigated the imbalance between the two groups by randomly down-sampling the no-recurrence cases. However, useful information may be discarded by such resampling, and the cases randomly selected may not represent accurately the rest of the patients. We are currently collecting recent data with a larger sample size to further validate and improve the current models. Last but not least, in the current ANN models, we only employed clinical factors that are readily available in clinical practice and imaging features that could be reliably identified with routine imaging exams, while subsequent relevant studies could accommodate more clinical and imaging factors that might influence the risk of recurrence in TIA and minor stroke patients. Automatic image analysis and image feature extraction by methods such as convolutional neural networks would help in establishing more intelligent models for risk stratification of such patients.

## Conclusion

Under the modern stroke service system, timely attention and management for TIA and minor stroke patients are becoming more readily available, which has significantly reduced the risk of stroke relapse in these patients. However, certain subgroups of patients are still at a high risk of subsequent disabling stroke, who may not be accurately identified with conventional risk predicting scores. Therefore, a more accurate and intelligent risk prediction strategy is needed. The ANN approach has advantages over conventional statistical methods or risk prediction scores that it could account for relationships between the independent variables, reflect complex relationships between continuous and categorical independent variables and the outcome, and quantify the weights of independent variables regarding their impact upon the outcome. The current pilot study indicated that ANN may yield a novel and effective method in risk stratification of TIA or minor stroke patients. Further studies are warranted for verification and improvement of such ANN models.

## Author Contributions

XL and JL made substantial contributions to the conception and design of the study. JY, YS, KW, and TL made substantial contributions to the acquisition of data. WZ, KC, WD, and QQ made substantial contributions to the analysis of data. KC, XL, WZ, and WD contributed to the interpretations of data. KC drafted the first version of the manuscript and XL made valuable revisions. All the authors revised the draft for intellectual content, gave their final approval of the final version for publication, and agreed to be accountable for all aspects of the work.

### Conflict of Interest Statement

The authors declare that the research was conducted in the absence of any commercial or financial relationships that could be construed as a potential conflict of interest.
