# Supplementary material for: Early Identification of High-Risk TIA or Minor Stroke Using Artificial Neural Network
Source: Front Neurol. 2019 Mar 1;10:171. doi: 10.3389/fneur.2019.00171 (PMC6405505; doi:10.3389/fneur.2019.00171)
Supplement: Supplementary file 1 [file Table_1.DOCX]

**Supplemental Table S1**. Definition and data type of independent variables in the ANN models

| Variables | Definition (if applicable) | Data type | |
| --- | --- | --- | --- |
| Age |  | Discrete data |  |
| Sex |  | Categorical data (male or female) |  |
| History of hypertension | Diagnosed prior to the index minor stroke/TIA or taking agents upon admission | Categorical data (yes or no) |  |
| History of diabetes mellitus |  |  |  |
| History of dyslipidemia |  |  |  |
| History of atrial fibrillation |  |  |  |
| History of ischemic stroke |  |  |  |
| History of TIA |  |  |  |
| History of ischemic heart disease |  |  |  |
| Current smoker |  | Categorical data (yes or no) |  |
| Unilateral weakness |  | Categorical data (yes or no) |  |
| Slurring speech |  |  |  |
| Duration of the neurological symptoms for transient deficits |  | Categorical data (3 categories: ≤10min, 11-60min and ＞60 min) |  |
| Systolic blood pressure | Systolic and diastolic blood pressure at admission | Continuous data |  |
| Diastolic blood pressure |  |  |  |
| NIHSS at admission |  | Discrete data |  |
| Premorbid mRS |  | Discrete data |  |
| Large artery stenosis | Presence of extracranial arterial stenosis and/or intracranial arterial stenosis | Categorical data (yes or no) |  |
| New infarct | Acute/subacute hypodensity on CT or hypertensity on T2 or diffusion weighted MRI | Categorical data (yes or no) |  |

Abbreviations: TIA indicates transient ischemic attack; NIHSS indicates National Institute of Health Stroke Scale; mRS indicates modified Rankin Scale.
